# Detection of Extremely Low Concentrations of Biological Substances Using Near-Field Illumination

**DOI:** 10.1038/srep39241

**Published:** 2016-12-19

**Authors:** Masato Yasuura, Makoto Fujimaki

**Affiliations:** 1 Electronics and Photonics Research Institute, National Institute of Advanced Industrial Science and Technology (AIST), 1-1-1 Higashi, Tsukuba, Ibaraki 305-8565, Japan

## Abstract

An external force-assisted near-field illumination biosensor (EFA-NI biosensor) detects a target substance that is propelled through an evanescent field by an external force. The target substance is sandwiched between an antibody coupled to a magnetic bead and an antibody coupled to a polystyrene bead. The external force is supplied by a magnetic field. The magnetic bead propels the target substance and the polystyrene bead emits an optical signal. The detection protocol includes only two steps; mixing the sample solution with a detection reagent containing the antibody-coated beads and injecting the sample mixture into a liquid cell. Because the system detects the motion of the beads, the sensor allows detection of trace amounts of target substances without a washing step. The detection capability of the sensor was demonstrated by the detection of norovirus virus-like particles at a concentration of ~40 particles per 100 μl in contaminated water.

Immunoassay-based methods for detection of biological substances, such as enzyme-linked immunosorbent assays and immunochromatography, have been widely used in various applications^1^,^2^,^3^. These methods, referred to as “sandwich assays”, employ the formation of a “sandwich” of the target substance between two different antibodies^4^,^5^. One of the antibodies captures the target substance on a sensing substrate, and the other provides a label. Such detection methods simultaneously exhibit high sensitivity and specificity. To achieve high sensitivity, a variety of electrical, mechanical, and optical sensors have been developed, such as the field effect transistor type sensor^6^, the microelectric-mechanical system type sensor^7^, and the surface plasmon resonance sensor^8^. They can detect changes in physical properties at the surface of a sensing substrate with high sensitivity. These sensing methods are highly sensitive because the capturing antibody concentrates target substances on a surface and the second labeled antibody enhances the signal. However, sensors that utilize surfaces have a serious disadvantage: non-specific adsorption of contaminants, which decreases their sensitivity and specificity^9^. To reduce non-specific adsorption, countless blocking and washing protocols have been developed, and various blocking and washing reagents are currently available. However, complete suppression of non-specific adsorption has not been achieved. To detect biomolecules at extremely low concentrations, a minimum volume of the sample solution must be applied so that the sample solution does contain the target substance. Thus, for sample solutions with lower concentrations, higher volumes must be applied.

An ideal immunosensor that can overcome the limitations of the current methods should have a wide effective sensing area and detect trace amounts of target substances in the presence of non-specific adsorption. In this study, we developed an “external force-assisted near-field illumination biosensor” (EFA-NI biosensor). The EFA-NI biosensor detects target substances that are propelled through an evanescent field by an external force. Candidates for the application of external force include magnetic force, electric force, and gravity. In the work described in the present report, we employed magnetic force as the external force. The sensing area of the EFA-NI biosensor is not a surface, but a free space near the surface. Using “moving signals” for detection, signal from the target substance can easily be distinguished from noise. Thus, the EFA-NI biosensor has the advantages, but not the disadvantages, of surface-based sensing methods.

## Results

### EFA-NI biosensor

The EFA-NI biosensor utilizes an enhanced electric field created by using near-field optics. It has been reported that a layered waveguide structure can generate a stronger and thicker enhanced electric field^10^,^11^,^12^. Therefore, a sensor chip with a 36-nm thick Si layer and 364-nm thick SiO_2_ layer placed on a 0.725-mm thick SiO_2_ substrate was employed in the present system. When S-polarized light is transmitted on to the sensor chip through a prism that is optically attached to the sensor chip at an incident angle of 67.6°, an enhanced electric field with a central wavelength of 644 nm is generated on the surface of the sensor chip. Figure S1 shows the calculated intensity of the electric field generated around the surface of the sensor chip under irradiation with S-polarized 644-nm light. The surface was assumed to be immersed in water. The intensity of the electric field at the sensor surface is 124 times stronger than that of the incident light. The intensity decreases with an increase in the distance from the surface. At a distance of 1200 nm from the surface, the intensity of the electric field is almost equal to that of the incident light.

**Figure 1:**
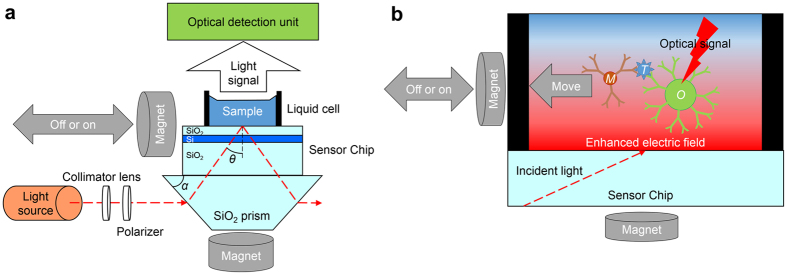
Configuration of the EFA-NI biosensor system. (**a**) Schematic of the sensor setup. A Xe lamp provides incident light via the optical fiber, collimator lens, and polarizer to the trapezoidal prism parallel to the sensor chip. The optical detection unit is a microscope equipped with an objective lens with a magnification of 5x. (**b**) Schematic showing the detection mechanism of the sensor. The particles *T, M*, and *O* are the target substance, the magnetic bead, and the bead for optical signal, respectively. The region marked with the red gradient is the region where the enhanced electric field is generated.

Figure 1a shows the setup of the EFA-NI biosensor. The sensor chip is placed on the bottom surface of a trapezoidal SiO_2_ glass prism with a bottom angle, *α*, of 32°. The surface of the sensor chip is illuminated with light from a Xe lamp through an optical fiber, a collimator lens, a polarizer, and the prism. The incident light is S-polarized. As shown in Fig. 1a, the light is transmitted on to the prism parallel to the sensor chip, and the incident angle, *θ*, becomes 67.6° at *λ* = 644 nm. A microscope equipped with an objective lens with a magnification of 5x and a cooled charge-coupled device (CCD) camera constitute the optical detection unit, as shown in Fig. 1a. The field of view of the system is approximately 2.5 mm × 2.0 mm. A 5-mm thick silicone rubber plate with an 8-mm diameter through-hole is placed on the sensor chip as a liquid cell. Two neodymium magnets (remanent flux density: 1.13 T) are used to apply the external magnetic force. One is placed under the prism while the other is placed next to the sensor chip and can be mechanically moved closer to the sensor chip. Figure 1b provides a schematic, detailing the detection mechanism of the sensor. The target substance, *T,* is sandwiched between an antibody on a magnetic bead, *M,* and another antibody on a bead, *O,* that provides an optical signal. The complex of the target substance, *T,* and the beads, *M* and *O,* is pulled toward the sensor chip surface by the magnet placed under the prism. This magnet pulls the complex into the enhanced electric field. The other magnet pulls the complex in a lateral direction. The two external forces combine to produce an optical signal indicating the presence of the complex. The signal moves laterally.

### Detection of normal mouse IgG

A normal mouse immunoglobulin G (NMIgG) was selected as the target substance of a proof-of-concept experiment. We examined the detection of NMIgG by the EFA-NI biosensor using magnetic beads (MBs) coated with a rabbit anti-mouse IgG and optical signal beads coated with a donkey anti-mouse IgG. The diameter of the MBs was 25 nm. In order to reduce noise, the MBs should be small enough and not emit optical signals. We observed the MBs with the EFA-NI biosensor and confirmed that the MBs did not emit detectable optical signals. The optical signal beads were 500-nm polystyrene beads (PSBs), and light scattered by the PSBs was used as the optical signal. The PSBs should also be as small as possible, because a smaller particle can be moved faster with less force. However, smaller PSBs emit less optical signals. The 500-nm PSBs were the smallest among the beads that we tested, the light scattered by which was strong enough to be observed with a video from the CCD camera of the system. A suspension of MBs at 100 particles/μl and 100 particles/μl PSBs was prepared as a “detection reagent”. Sample solution (100 μl) and the detection reagent (100 μl) were mixed, incubated for 15 min at room temperature (approximately 25 °C), and 100 μl of the mixture was injected into the liquid cell. Samples containing NMIgG, MBs, and PSBs are expected to form MB-NMIgG-PSB complexes.

The surface of the EFA-NI biosensor chip was modified to suppress non-specific adsorption. The modification was intended not only to suppress non-specific adsorption of contaminants but also to prevent adsorption of MB-NMIgG-PSB complexes on the sensor chip surface. If the complex is adsorbed non-specifically on the surface, the complex cannot be propelled by the magnetic force and therefore cannot be detected by the sensor. In fact, particle movement was almost absent when a biosensor chip without surface modification was used.

**Figure 2:**
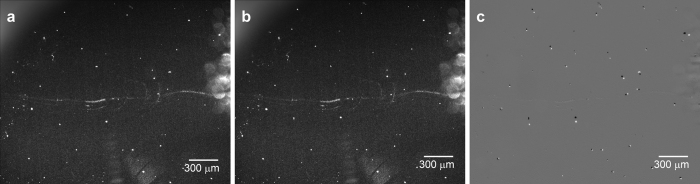
Photographs of the sensor chip surface in the EFA-NI biosensor (**a**) before magnetic field application from the side of the sensor chip, (**b**) 60 s after magnetic field application. (**c**) Difference between (**a**,**b**). The white spots are the bright spots observed only in (**a**), while the black spots are those observed only in (**b**).

Figure 2 shows images captured during the detection of NMIgG. The concentration of NMIgG in the sample solution was 10 fg/ml, which means that the 100 μl of the mixture contained approximately 2000 NMIgG particles. The bright white spots in the images are scattered light signals from the MB-NMIgG-PSB complexes and contaminants in the enhanced electric field. Figure 2a displays an image captured before the application of magnetic field from the side of the sensor chip. This image was captured with an exposure time of 10 s. It was observed on the video that some of the bright spots were moving randomly in different directions, while some others were staying in the same positions (data not shown). Figure 2b shows an image captured 60 s after the application of the magnetic field from the side of the sensor chip. The magnetic force was applied from the upper side of the figure. The exposure time was 10 s, and during this period, the magnetic field from the side was off. Figure 2c shows the difference between Fig. 2(a,b). The white spots are the bright spots observed only in Fig. 2a, while the black spots are those observed only in Fig. 2b. The bright spots that did not change the position are absent in Fig. 2c. There are 45 white spots in Fig. 2c, among which, 12 spots were moving before the application of the magnetic field and did not change direction on the application of the magnetic field, indicating that the other 33 spots represent MB-NMIgG-PSB complexes. As can be seen in Fig. 2, the size of the spots was not uniform. The larger spots would be aggregates of the MBs, NMIgG, and PSBs. The number of NMIgG particles was about 2000 in the solution, whereas the detected spots were only 33. This would be due to the formation of aggregates.

### Detection of norovirus virus-like particles

A norovirus virus-like particle (VLP)^13^,^14^ of GII.4 norovirus was selected as the target substance in this experiment. Norovirus infection occurs across the globe and causes emesis, diarrhea, and fever^15^,^16^,^17^. Norovirus is highly infectious, and the minimum infectious dose has been reported to be as low as <20 viral particles per person^18^,^19^. Hence, to control norovirus infection, a detection method must be able to detect extremely low numbers of viral particles in a sample. Currently, polymerase chain reaction (PCR) is the main method of norovirus detection and identification, but in order to prevent false positives owing to contamination, double-checking by an immunoassay is desirable. However, the sensitivity of immunoassays, such as immunochromatography, is too low to be an alternative for PCR^20^,^21^.

In the present research, we examined the detection of norovirus VLPs by the EFA-NI biosensor, using MBs coated with an anti-norovirus monoclonal antibody (12A11)^22^ and PSBs coated with an anti-norovirus polyclonal antibody. A suspension containing MBs at 100 particles/μl and PSBs at 10 particles/μl was prepared as the “detection reagent.” Sample suspension (100 μl) and detection reagent (100 μl) were mixed, incubated for 15 min at room temperature (approximately 25 °C), and 100 μl of the mixture was injected into the liquid cell. The surface of the sensor chip was modified to suppress non-specific adsorption.

**Figure 3:**
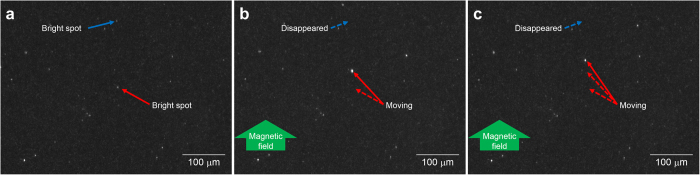
Photographs of the sensor chip surface in the EFA-NI biosensor at (**a**) t = 0 s (at the initiation of magnetic field application from the side of the sensor chip), (**b**) t = 9 s, and (**c**) t = 17 s after the initiation of magnetic field application. The red and blue arrows indicate a bright spot moving within the image and a bright spot that disappeared from the image, respectively. The magnetic field was applied from the upper side of the figures.

Figure 3 shows images captured during detection of VLPs. The VLP concentration of the suspension was 10 fg/ml, which means that the 100 μl of the mixed suspension contained approximately 40 VLPs. The bright white spots in the images are scattered light signals from the MB-VLP-PSB complexes and contaminants in the enhanced electric field. We had previously confirmed that the MBs were small enough not to be observed as bright spots. Figure 3(a–c) show images captured at t = 0 s, t = 9 s, and t = 17 s after initiation of magnetic field application, respectively, from the side of the sensor chip. One bright spot was observed to move in the direction of the magnetic field. This spot was the complex and is indicated by the red arrow in Fig. 3. Another bright spot, which was removed from the field of view by the magnetic force, was also considered to be a complex and is indicated by the blue arrow. The other bright spots, which stayed in the same position, are contaminants or PSBs that did not form complexes. Supplementary Video 1 shows video footage corresponding to the images shown in Fig. 3, as well as the full display version of the movie. As shown in the full display version of Supplementary Video 1, the EFA-NI biosensor successfully detected three signal spots for approximately 40 VLPs in the 100-μl suspension.

VLP detection was performed with a solution containing VLPs at concentrations ranging from 0–10 pg/ml (approx. 0–40000 VLPs in 100 μl of mixed solution). No moving or disappearing bright spots were observed in the solution that did not contain VLPs. The number of moving and disappearing bright spots observed when the magnetic field was applied to solutions containing 0.01, 0.1, 1, and 10 pg/ml of VLPs was 2.7 ± 0.6, 3.7 ± 1.5, 3.3 ± 1.5, and 7.3 ± 3.2, respectively. These results show the standard deviations, when n was 3 for each of the different samples. This result indicates that the number of moving or disappearing bright spots observed is always much smaller than that of the VLPs in the sample solution, and it does not increase in direct proportion to the number of VLPs in the solution.

There are two possible explanations for these results. First, the field of view of the microscope is 10 times smaller than the area of the sensor chip surface within the liquid cell. This means that the majority of the MB-VLP-PSB complexes are outside the field of view. This problem could be solved by optimizing the microscope setup and the size of the liquid cell. Second, not all of the VLPs would necessarily have formed complexes. Some of the VLPs might have conjugated with only MBs or PSBs. These “wrong complexes” do not emit a signal. This could also be a reason why only a few complexes were observed in mixed suspensions that contained 400 or more VLPs. In the experiment, the detection reagent was adjusted for the detection of VLPs at extremely low concentrations, such as 40 particles per 100 μl. The bead concentration was adjusted to be as low as possible, to avoid formation of wrong complexes. Therefore, if the number of VLPs was increased to 10^3^ particles per 100 μl, some of the VLPs would not form complexes, and complex formation would be saturated, because it would be limited by the number of PSBs. If the number of VLPs were increased to 10^4^ particles per 100 μl or more, the number of complexes formed might decrease, owing to a shortage of beads. This problem could be solved by developing an optimal detection reagent, an optimal pair of antibodies, and beads. However, the development of the optical detection reagent is not so simple. Formation of aggregates also reduces the number of complexes observed. As shown in Fig. 2, larger spots, which would be aggregates of the MBs, NMIgG, and PSBs, were observed. The moving and disappearing bright spots observed in the VLP detection experiments may contain two or more VLPs. If the concentration of the beads is increased, it would result in the formation of larger aggregates. Supplementary Video 2 shows video footage observed during the VLP detection performed with a suspension containing approximately 4000 VLPs in 100 μl of sample mixed with 100 μl of detection reagent composed of MBs at 1000 particles/μl and PSBs at 100 particles/μl. A moving aggregate could be clearly observed in the video. For quantitative analysis using the EFA-NI biosensor, not only the number of the beads but also the number of the antibodies on the beads should be controlled to suppress the formation of aggregates.

So far, the lower detection limit of the present system is 40 particles/100 μl. If an ideal detection reagent could be developed, the upper detection limit will only be limited by the number of pixels of the camera and the pixel size of the signal of the PSBs, which were 3380 × 2704 and about 15 pixels in the present experiment, respectively. This means that the camera can simultaneously observe approximately 10^5^ particles. Since the camera observes 10% of the surface area of the sensor chip within the liquid cell, the upper detection limit of the present system is in the order of 10^6^ particles/100 μl.

### Detection of norovirus VLPs in treated wastewater

**Figure 4:**
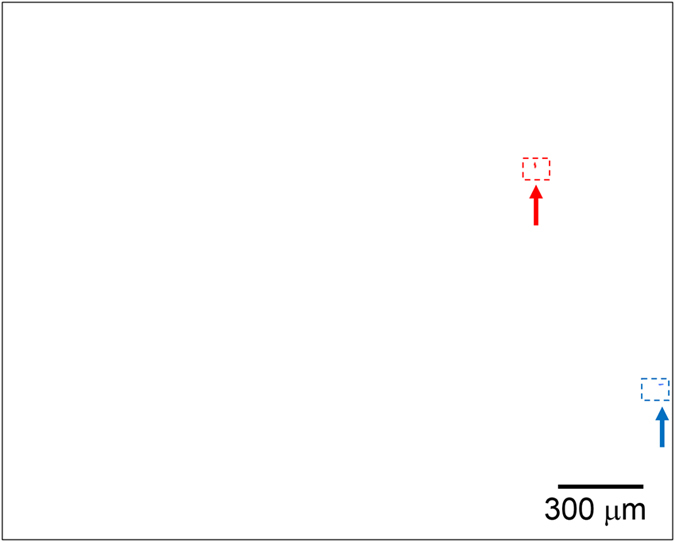
Detection of VLPs in secondary treated wastewater. The blue and red dots indicate bright spots observed only before and only after application of the magnetic force, respectively. Bright spots observed both before and after applying the magnetic field were erased.

We performed VLP detection in secondary treated municipal wastewater. The treated wastewater was examined using a nanoparticle tracking system (Nanosight LM10, Nanosight Ltd., Malvern, UK) and was found to contain particles of 100–500 nm diameter at a concentration of ~3 × 10^9^ particles/ml of contaminants. VLPs were added to the treated wastewater to 20 fg/ml concentration. Treated wastewater (100 μl) and detection reagent (100 μl) were mixed and incubated for 15 min at room temperature (approximately 25 °C). The mixture (200 μl) contained ~80 VLPs and ~3 × 10^8^ contaminants. The liquid cell was filled with the mixture. Figure 4 shows the detection results obtained by the EFA-NI biosensor. Since there were many bright spots because of the non-specific adsorption of contaminants, the image was treated to make the ‘moving signals’ easier to recognize. Figure 4 has white background. Bright spots observed only before and only after the application of the magnetic field can be seen as blue and red dots, respectively. Bright spots observed both before and after application of the magnetic field were erased. The blue and red dots indicate MB-VLP-PSB complexes that were removed from or brought into the field of view by the magnetic force, respectively. As shown in Fig. 4, the sensor recognized two signal spots for ~80 VLPs in the 200-μl sample. This result indicates that the sensor retains high detection sensitivity, even in a sample containing contaminants. It should also be emphasized that no washing process was applied during the detection process. Supplementary Video 3 corresponds to the image shown in Fig. 4.

## Discussion

It has been reported^16^ that norovirus is the cause of 18% of the cases of acute gastroenteritis. Because the minimum infectious dose is less than a few tens of viral particles^18^,^19^, it is imperative to develop a highly sensitive system for norovirus detection. Although immunochromatography is convenient, its sensitivity is inadequate. PCR, another commonly used method, requires an extremely clean environment to prevent false positive results. The EFA-NI biosensor is a novel immunoassay with extremely high sensitivity and specificity. The sensor can detect VLPs at a concentration of 40 particles per 100 μl. The use of an enhanced electric field at a distance of ~1 μm from the sensor chip makes it possible to efficiently generate scattered light from the 500-nm PSBs and to detect floating MB-VLP-PSB complexes near the surface of the sensor. Using strong scattered light as the optical signal, the sensor can detect the signal from the complex in a wide field of view at several frames per second, yielding high sensitivity and specificity. The EFA-NI biosensor does not need any flow systems, such as pumps, tubes, or tanks, which means that sensor setup is much simpler than the current particle detection systems that use flow systems, such as flow cytometers.

In addition to the merits of the detection system itself, the EFA-NI biosensor provides detection abilities superior to those of conventional techniques. This sandwich immunoassay requires only two steps: “pre-mixing” of the sample with the detection reagent and injection of the mixture into the liquid cell, prior to recording the measurement. The sensor does not require washing. During the “pre-mixing” process, antigen-antibody interactions occur in solution and not on the surface. Therefore, the efficiency of the interaction is much higher than that in surface reaction methods. Hence, reaction time is expected to be shortened. Since the sensor does not rely on adsorption of target substances on the chip surface, the sensor chip can be used repeatedly.

As shown above, the EFA-NI biosensor is a high-sensitivity immunoassay-based biosensor, which distinguishes signal from noise by detecting the motion of immunocomplexes that are propelled through an evanescent field by an external force. Despite the simplicity of the method, the sensor can detect extremely low concentrations of viruses, even in a contaminated water sample. The EFA-NI biosensor is not suitable for detection of substances at high concentrations. In such a case, dilution is essential to lower the concentration of the sample substance as well as that of contaminants. Although quantitative detection and a wider dynamic range require further developmental work, the EFA-NI biosensor shows promise to become a core biosensing technique in the near future.

## Methods

### Calculation of the intensity of the enhanced electric field

The transfer matrix method for a stratified medium according to the Fresnel equations^23^ was used to calculate the intensity of the enhanced electric field. The complex refractive indices of the SiO_2_ glass and water were adapted from a previous report^24^. The complex refractive index of the single-crystalline Si was adapted from a previous report^25^.

### Detection system

The dimensions of the sensor chip used in this study were 14 mm × 18 mm × 0.725 mm. The sensor chip had a layered structure, with a 36-nm Si layer and a 364-nm SiO_2_ layer on a SiO_2_ substrate (Shin-Etsu Chemical Co., Ltd., Tokyo, Japan). The method of fabrication of the sensor chip has been described in detail previously^26^. The SiO_2_ surface of the sensor chip was modified with a self-assembled monolayer (SAM) using N-[3-(triethoxysilyl)propyl]-3,6,9,12-tetraoxatridecanamide to prevent non-specific adsorption^27^. A trapezoidal silica glass prism with a bottom angle *α* of 32° was used as the prism, as shown in Fig. 2. The sensor chip was attached to the prism via a refractive index matching liquid (Code 50350, *n* = 1.4587 ± 0.0005 at 589.3 nm, Cargille-Sacher Laboratories Inc., NJ, USA). A wavelength-tunable Xe Lamp (SM-10YN, λ = 300–1100 nm, 150 W, Bunkoukeiki Co., Ltd., Tokyo, Japan) was used as the light source. Collimated and s-polarized white light was transmitted from the light source onto the prism, parallel to the sensor chip surface via an optical fiber (600 μm*ϕ* core, Thorlabs Japan Inc., Tokyo, Japan), a collimator lens (74-UV, focus 10 mm, Ocean Optics, Inc., FL, USA), and a linear polarizer (#89-602, λ = 300–2700 nm, Edmund Optics Japan, Tokyo, Japan). The magnetic field was applied using neodymium magnets (remanent flux density: 1.13 T, ASONE, Osaka, Japan). An upright microscope equipped with an objective lens with 5x magnification (M Plan Apo NIR 5×, Mitutoyo Co., Kanagawa, Japan) and a cooled CCD camera (BU-59LIR, Bitran Co., Saitama, Japan) was used as the optical detection unit.

### Preparation of the detection reagent for normal mouse IgG

Two kinds of anti-mouse antibodies, donkey anti-mouse IgG (H + L) (Jackson ImmunoResearch Laboratories, Inc., PA, USA) and rabbit anti-mouse IgG (H + L) (Jackson ImmunoResearch Laboratories, Inc., PA, USA), were used in the experiments. These antibodies were modified with biotin using the biotin labeling kit (Dojindo Laboratories, Kumamoto, Japan). The rabbit anti-mouse IgG-coated MB suspension with a concentration of ~10^5^ particles/μl was prepared by mixing 200 μl of biotinylated rabbit anti-mouse IgG at a concentration of 500 fg/μl and 200 μl of streptavidin-conjugated 25 nmϕ MBs (MP25-AV, Nanocs Inc., NY, USA) at a concentration of ~10^5^ particles/μl. The donkey anti-mouse IgG-coated PSB suspension at a concentration of ~10^5^ particles/μl was prepared by mixing 200 μl of the biotinylated donkey anti-mouse IgG with a concentration of 500 fg/μl and 200 μl streptavidin-conjugated 500 nmϕ PSBs (PS500-AV, Nanocs Inc., NY, USA) at a concentration of ~10^5^ particles/μl. The antibodies and the beads were incubated overnight at 4 °C to permit sufficient reaction time. The detection reagent was prepared by mixing 1 μl of the MB suspension, 1 μl of the PSB suspension, and 998 μl of Milli-Q water.

### Detection of normal mouse IgG

Normal mouse IgG (Wako Pure Chemical Industries, Ltd., Osaka, Japan) was used as the target substance to be detected. A mixture of the sample solution containing NMIgG and the detection reagent was injected into the liquid cell of the sensor. Magnetic field was applied using a neodymium magnet placed under the prism to attract the MB-NMIgG-PSB complex on to the surface of the sensor chip. The magnetic field was always applied during detection. The magnet placed next to the sensor chip was moved closer to the sensor chip to begin the application of the magnetic field, in order to move the complex laterally. The surface of the sensor chip was monitored using a cooled CCD camera to capture two-dimensional micrographs. Two micrographs were taken by the CCD camera before and after the application of the magnetic field. The exposure time was 10 s.

### Preparation of the detection reagent for norovirus virus-like particles

The anti-norovirus monoclonal antibody used in this experiment was 12A11, which showed intra-GII cross-reactivity^22^. To prepare the anti-norovirus polyclonal antibody, hyperimmune sera to 13 genotypes of VLPs, including GI.1, GI.2, GI.3, GI.4, GI.6, GII.1, GII.3, GII.4, GII.5, GII.6, GII.7, GII.12, and GII.13, were prepared in rabbits and the 13 antisera were mixed in equal volumes. The IgG molecules from the mixed antisera, purified with Protein G Sepharose, were used as the polyclonal antibody. These antibodies were modified with biotin using the biotin labeling kit (Dojindo Laboratories, Kumamoto, Japan). The monoclonal antibody-coated MB suspension with a concentration of 10^5^ particles/μl was prepared by mixing 10 μl of biotinylated monoclonal antibody solution at a concentration of 1 ng/μl and 100 μl of streptavidin-conjugated 25 nm*ϕ* MB suspension (MP25-AV, Nanocs Inc., NY, USA) at a concentration of 10^6^ particles/μl, and 890 μl of Milli-Q water. The polyclonal antibody-coated PSB suspension at a concentration of 10^4^ particles/μl was prepared by mixing 1 μl of the biotinylated polyclonal antibody solution at a concentration of 1 ng/μl, 100 μl streptavidin-conjugated 500 nm*ϕ* PSB suspension (PS500-AV, Nanocs Inc., NY, USA) at a concentration of 10^5 ^particles/μl, and 899 μl of Milli-Q water. The antibodies and the beads were incubated overnight at 4 °C to permit sufficient reaction time. The detection reagent was prepared by mixing 1 μl of the MB suspension, 1 μl of the PSB suspension, and 998 μl of Milli-Q water.

### Detection of norovirus virus-like particles

Strain Narita 104 VLP (r104: genogroup II, genotype 4 [GII.4]), prepared by infecting subconfluent Tn5 insect cells with recombinant baculoviruses, as described previously^14^, was used as the target substance to be detected. The concentration of the original solution was determined by a method described previously^28^. A mixture of the sample solution containing VLPs and the detection reagent was injected into the liquid cell of the sensor. Magnetic field was applied using a neodymium magnet placed under the prism to attract the MB-VLP-PSB complex onto the surface of the sensor chip. The magnetic field was always applied during detection. The surface of the sensor chip was monitored using a cooled CCD camera to capture two-dimensional micrographs or videos. The exposure time of the burst capture was 300 ms. The magnet placed next to the sensor chip was moved closer to the sensor chip during monitoring by the CCD camera, to begin application of the magnetic field, in order to move the complex laterally.

## Additional Information

**How to cite this article:** Yasuura, M. and Fujimaki, M. Detection of Extremely Low Concentrations of Biological Substances Using Near-Field Illumination. *Sci. Rep.* **6**, 39241; doi: 10.1038/srep39241 (2016).

**Publisher's note:** Springer Nature remains neutral with regard to jurisdictional claims in published maps and institutional affiliations.

## Supplementary Material

Supplementary Information

Supplementary Video 1

Supplementary Video 2

Supplementary Video 3
