# Supplementary material for: Detection of Extremely Low Concentrations of Biological Substances Using Near-Field Illumination
Source: Sci Rep. 2016 Dec 19;6:39241. doi: 10.1038/srep39241 (PMC5171845; doi:10.1038/srep39241)
Supplement: Supplementary Information [file srep39241-s1.pdf]

Supplementary Information

Detection of Extremely Low Concentrations of Biological Substances Using Near-Field Illumination

Masato Yasuura[1] and Makoto Fujimaki[1,*]

[1]Electronics and Photonics Research Institute, National Institute of Advanced Industrial Science and Technology (AIST), 1-1-1 Higashi, Tsukuba, Ibaraki 305-8565, Japan

[*]Correspondence to m-fujimaki@aist.go.jp

Table of contents

Figure S1: Calculation of the intensity of the electric field generated near the surface of the sensor chip by irradiation with 644-nm light at an incident angle of 67.6°. (p. S3)

Supplementary Video 1: Detection of 10 fg/ml VLP in the subsection "Detection of norovirus virus-like particles". (Separate file)

Supplementary Video 2: Aggregated complexes in the experiment to detect 1000 fg/ml VLP. (Separate file)

Supplementary Video 3: Detection of 10 fg/ml VLP in secondary treated wastewater in the subsection "Detection of norovirus VLPs in treated wastewater". (Separate file)

Figure S1

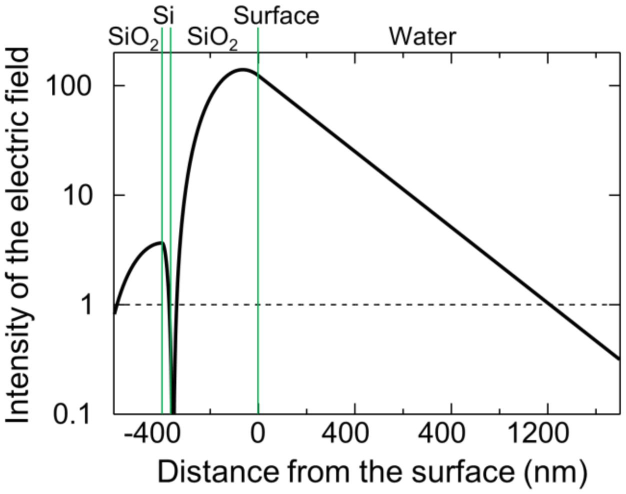

Figure S1. Calculation of the intensity of the electric field generated near the surface of the sensor chip by irradiation with 644-nm light at an incident angle of 67.6°. The intensity was normalized to the intensity of the incident light. On the x axis, 0 indicates the surface of the sensor chip. The green lines indicate the boundary between the layers. The region x > 0 is assumed to be water.

Text summaries of each video

Supplementary Video 1

This video shows footage corresponding to the images shown in Fig. 3 in the subsection "Detection of norovirus virus-like particles". Three signal spots were observed.

Supplementary Video 2

This video shows a big bright spot of aggregated complexes, which could be moved by the magnetic field. The bright spot moved to the upper side and lower side of the screen alternatively with the magnetic field application from each side.

Supplementary Video3

This video shows footage corresponding to the images shown in Fig. 4 in the subsection "Detection of norovirus VLPs in treated wastewater". Two signal spots were observed.

Legends of each video

Supplementary Video 1

Video footage of the sensor chip surface observed by the EFA-NI biosensor during the detection of 10 fg/ml VLP in the subsection "Detection of norovirus virus-like particles". The magnetic field was applied from the upper side of the screen. Two bright spots moving in the direction of the magnetic field and one bright spot that disappeared with the application of magnetic field are observed. Blinking or slight random movement of the spots on the screen is due to Brownian

motion.

Supplementary Video 2

Video footage of the sensor chip surface observed by the EFA-NI biosensor during the detection of 1000 fg/ml VLP. The detection reagent was composed of 1000 particles/μl MBs and 100 particles/μl PSBs. The magnetic field was applied from the upper side and the lower side of the screen alternatively. One big bright spot moving in the direction of the magnetic field with the application of the magnetic field was observed. The big bright spot indicates the presence of aggregates.

Supplementary Video 3

Video footage of the sensor chip surface observed by the EFA-NI biosensor during the detection of 10 fg/ml VLP, in secondary treated wastewater in the subsection “Detection of norovirus VLPs in treated wastewater”. The magnetic field was applied from the upper side of the screen. One bright spot moving in the direction of the magnetic field and one bright spot that disappeared with the application of magnetic field are observed. This result indicates that the EFA-NI biosensor can detect VLP even in contaminated water.
